# Pro‐inflammatory endothelial cell activation mediated by protofibrillar tau leads to loss of barrier function and integrity

**DOI:** 10.1002/alz.092830

**Published:** 2025-01-03

**Authors:** Roberto A Guzman‐Hernandez, Silvia Fossati

**Affiliations:** ^1^ Lewis Katz School of Medicine, Temple University, Philadelphia, PA USA; ^2^ Alzheimer’s Center at Lewis Katz School of Medicine, Temple University, Philadelphia, PA USA

## Abstract

**Background:**

Neurofibrillary tangles formed by hyperphosphorylated tau aggregates in the brain are one of the classical hallmarks of Alzheimer’s Disease (AD). Tau aggregates have been shown to elicit cytotoxicity, leading to overall neuronal loss and cognitive decline in AD. These aggregates can be transmitted from neurons and glial cells to other brain cells through a process known as tau spreading, and ultimately reach the endothelial cells (ECs) lining the vessel walls, thus, causing dysfunction of the neurovascular unit (NVU), a complex multicellular system surrounding brain vessels. The NVU is extremely important for blood flow regulation, amyloid and tau clearance, and proper brain function, however, the mechanisms responsible for the effects of tau on ECs remain understudied. We hypothesize that protofibrillar tau mediates pro‐inflammatory EC activation tied to bioenergetic alterations, culminating in loss of barrier function.

**Method:**

Immortalized (D3) and primary (hCECs) human brain microvascular ECs were challenged with protofibrillar 1N4R tau. Trans‐endothelial electrical resistance (TEER) was measured by the ECIS Zθ system as an *in‐vitro* model of the BBB. Cytokine production was assessed using a V‐Plex Neuroinflammatory Panel 1 (MSD). IL‐4 release was determined in cell supernatant. Immunocytochemical analysis of junction protein (ZO‐1 and VE‐Cadherin) expression and western blotting for EC activation markers (VCAM‐1 and ICAM‐1) and junction proteins was performed. EC bioenergetics were measured by Seahorse Extracellular Flux Analyzer and EC death was determined, as well as caspase activation.

**Result:**

Reduced TEER was observed with protofibrillar tau, independently of EC death. VCAM‐1 expression significantly increased, whereas junction protein expression was decreased. Aggregated tau caused an increase in glycolysis at 24h, which reverted at further timepoints, whereas mitochondrial ATP production increased. IL‐4 production was increased at 24 hours, with similar trends at earlier timepoints. Blocking IL‐4 activity partially rescued TEER and decreased VCAM‐1 expression.

**Conclusion:**

Our results suggest that tau protofibrils mediate pro‐inflammatory EC activation, coupled with metabolic alterations. These molecular changes can contribute to loss of barrier integrity, exacerbating cerebrovascular dysfunction.

